# AEN Suppresses the Replication of Porcine Epidemic Diarrhea Virus by Inducing the Expression of Type I IFN and ISGs in MARC-145 Cells

**DOI:** 10.3390/pathogens13010024

**Published:** 2023-12-27

**Authors:** Miao Luo, Jiale Ma, Xinming Pan, Xinqin Zhang, Huochun Yao

**Affiliations:** 1 College of Veterinary Medicine, Nanjing Agricultural University, Nanjing 210095, China; 2 MOE Joint International Research Laboratory of Animal Health and Food Safety, College of Veterinary Medicine, Nanjing Agricultural University, Nanjing 210095, China; 3 Key Laboratory of Animal Bacteriology, Ministry of Agriculture, Nanjing Agricultural University, Nanjing 210095, China

**Keywords:** PEDV, AEN, IFN, ISGs

## Abstract

Apoptosis-enhancing nuclease (AEN), which shares close evolutionary relationships with the interferon-stimulated gene 20 protein (ISG20) homologs in humans, is a member of the DEDDh exonuclease family. Numerous studies on various pathogens have identified the essential roles of ISG20 in inhibiting virus replication. However, the fundamental functions of AEN during viral infection remain largely unknown. This study discovered that AEN expression was significantly upregulated in MARC-145 cells infected with Porcine epidemic diarrhea virus (PEDV) strain 85-7. In contrast, the amount of AEN protein decreased as viral replication increased. It was found that PEDV nsp1 and nsp5 mediated the decrease in AEN production, suggesting that an increase in AEN was not conducive to virus replication. By comparing AEN and its exonuclease-inactive mutant AEN-4A, we determined that the antiviral activity of AEN was independent of its exonuclease function. qPCR analyses revealed that AEN and AEN-4A could induce a significant increase in the transcription levels of IFN-α, IFN-β, and ISGs (OASL, IFI44, IFIT2, ISG15, Mx1, Mx2), and that AEN-4A has a higher induction ability. Overexpression of AEN and AEN-4A in MARC-145 cells targeting IFN-β knockdown or IFN-deficient Vero cells showed reduced or a complete loss of antiviral activity of both, suggesting that AEN may activate the type I IFN immune response and promote the expression of ISGs, thereby inhibiting PEDV replication. Taken together, our data prove the novel mechanism of AEN-mediated virus restriction.

## 1. Introduction

Porcine epidemic diarrhea virus (PEDV) is an enveloped virus with a single-stranded positive-sense RNA genome that belongs to the genus *Alphacoronavirus* (α-CoV) [1]. The whole viral genome is approximately 28 kb, encoding two polyproteins (pp1a and pp1ab), four structural proteins (spike, S; envelope, E; membrane, M; and nucleocapsid, N) and an accessory protein (open reading frame 3, ORF3) [2,3]. PEDV can cause porcine epidemic diarrhea (PED), which is a highly contagious and severe enteric disease first recognized in England in 1971 [4], causing lethal watery diarrhea, vomiting, and high mortality in neonatal piglets, leading to enormous economic losses to the swine industry [5,6,7].

The IFN immune response is the first barrier of host defense against pathogen infection associated with multiple mechanisms and proteins [8]. So far, type I IFN has been widely studied among three identified IFN types (type I, II, and III). Type I IFN production is secreted extracellularly, binds to IFN receptors 1 (IFNAR1) and 2 (IFNAR2), and triggers the expression of IFN-stimulating genes (ISGs) via the JAK-STAT pathway, which has direct or indirect antiviral effects in host cells [9]. Our previous studies confirmed that IFN-β and the other ISGs (OASL, IFI44, IFIT2, ISG15, Mx1, Mx2) could inhibit PEDV replication [10,11]. Meanwhile, PEDV escaped the immune response by inhibiting the production of IFN-β [10]. Therefore, it is critical to understand the underlying mechanisms of PEDV replication and determine how PEDV manipulates the host immune response.

Apoptosis-enhancing nuclease (AEN) shares a high similarity with the exonuclease III and exhibits nuclear colocalization with apoptotic nuclease such as CAD and AIF after irradiation, thus it affects the cellular radiation response by enhancing apoptosis [12]. T Kawase et al. [13] verified that AEN targets both single- and double-stranded DNA and RNA in human, and induces apoptosis. In addition, AEN and BAX are the main targets in the induction of apoptosis in response to 2-Aminoanthracene exposure [14]. AEN, along with the closely related interferon-stimulated gene 20 protein (ISG20) and ISG20L2, belongs to the DEDDh exonuclease family that is named ISG20L1 in human [15]. The members of this family possess three characteristic exonuclease motifs (Exo I, Exo II, and Exo III) defined by five conserved amino acids, DEDD, and a histidine residue [16]. Numerous studies have shown that ISG20 can inhibit viral replication [17,18,19,20], such as influenza A virus [21], bluetongue virus [22], pseudorabies virus [23], and hepatitis B virus [24].

In this study, we verified that AEN and AEN-4A can significantly inhibit PEDV replication. AEN and AEN-4A can significantly promote the expression of type I IFN and induce the increase in transcription level of ISGs downstream of IFN in MARC-145 cells; and AEN-4A has a higher induction ability. However, AEN and AEN-4A lost their antiviral activity completely in IFN-deficient Vero cells, and targeted knockdown of IFN-β expression level in MARC-145 cells significantly weakened the antiviral effect of both, thus demonstrating that AEN relies on IFN immune response to exert its antiviral activity.

## 2. Materials and Methods

### 2.1. Cells, Viruses, and Plasmids

MARC-145 cells and Vero cells (African green monkey kidney cells) were cultured in Dulbecco’s modified Eagle’s essential medium (DMEM; Hyclone Laboratories, Logan, Utah, USA) supplemented with 10% fetal bovine serum (FBS; Gibco, Carlsbad, CA, USA) and 1% penicillin and streptomycin at 37 °C in a 5% CO_2_ incubator. The porcine epidemic diarrhea virus (PEDV) 85-7 used in this study was isolated previously in our laboratory and propagated in MARC-145 cells. PEDV CV777 was kindly provided by Prof. Xiang Mao. The *AEN* gene was amplified from the cDNA of MARC-145 cells and cloned into C-terminal p3 × FLAG-CMV-14, designated as Flag-AEN. The mutant plasmid Flag-AEN-4A, was prepared using site-directed mutagenesis. The PEDV nsp1 and nsp5 genes were amplified and cloned into N-terminal pCAGGS-HA, designated as HA-nsp1 and HA-nsp5. The primers for constructing the plasmids are shown in Table S1.

### 2.2. Antibodies and Other Reagents

Anti-Flag antibody (F1804) was purchased from Sigma-Aldrich (St. Louis, MO, USA). Anti-AEN antibody was purchased from Thermo Fisher Scientific (Waltham, MA, USA). Anti-HA antibody was purchased from Abmart (Shanghai, China). Anti-β-actin antibody (ab179467) was purchased from Abcam (Cambridge, UK). PEDV N polyclonal antibody was generated in our laboratory. Horseradish peroxidase (HRP)-conjugated goat anti-mouse IgG (H + L) and HRP-conjugated goat anti-rabbit IgG (H + L) antibodies were obtained from Cell Signaling Biotechnology (Danvers, MA, USA). The siRNA specifically designed to knock down *IFNB1* expression, as well as the control siRNA, were purchased from Genepharma (Shanghai, China).

### 2.3. Viral Infection and Titer Determination

Briefly, a confluent monolayer of Vero cells or MARC-145 cells were infected with 85-7 strain in DMEM without FBS by incubation at 37 °C for 1 h. The cells were then washed thrice with phosphate-buffered saline (PBS). The cells were cultured in maintenance medium (DMEM with 2% FBS) at 37 °C with 5% CO_2_. The cells were harvested when a cytopathic effect (CPE) of 80~90% was observed. The cell pellet underwent one freeze–thaw cycle and was then centrifuged at 10,000× *g* for 15 min at 4 °C. The supernatant was harvested and stored at −80 °C. The plaque formation assay was used to determine the virus titer. Briefly, 10-fold serial dilutions (10^1^–10^6^) of viruses were incubated in MARC-145 cells. Unbound viruses were washed away with PBS after 1 h of incubation at 37 °C. The plate was incubated at 37 °C with 5% CO_2_ for 2 to 3 days before adding an overlay medium (2% low-melting-point agarose in DMEM containing 2% FBS). Plaque-forming units (PFUs) per milliliter were calculated after the cells were stained with crystal violet (0.5 g of crystal violet dissolved in 10 mL of methyl alcohol, 40 mL of formaldehyde, and 50 mL of water).

### 2.4. RNA Extraction, Reverse Transcription PCR (RT-PCR), and Quantitative PCR (qPCR)

An RNAiso Plus reagent kit (Vazyme, Nanjing, China) was used to extract total RNA from PEDV-infected and uninfected MARC-145 cells. The qPCR was carried out to determine the transcription levels of the specific genes, using SYBR^®^ Premix Ex TaqTM II (Takara, Otsu, Shiga, Japan) on an ABI 7300 real-time PCR system (Foster City, CA, USA). Table S2 contains a list of the qPCR primers. The data were normalized to the β-Actin gene’s expression level. The relative quantities of mRNA accumulation were calculated using the 2^−ΔΔCt^ method. The reported values are from three independent tests.

### 2.5. Transient Transfection

The highly purified plasmids were extracted using an E.Z.N.A.^®^ Endo-Free Plasmid Mini Kit I (Omega Bio-tek, Norcross, GA, USA). First, 3 × 10^5^ MARC-145 cells were seeded into each well of a 12-well plate for 12 h before transfection. The corresponding plasmids were transfected into MARC-145 cells at a ratio of 1 μg DNA to 2 μL Lipofectamine 2000 (Invitrogen, Carlsbad, CA, USA). Other procedures were carried out according to the manufacturer’s instructions.

### 2.6. Short Interfering RNA (siRNA) Knockdown of the IFNB1 Gene

The mRNA derived from the *IFNB1* gene was depleted by siRNA-mediated gene silencing on MARC-145 cells. A siRNA (siIFNB1: F: 5′-GCCUCAAGGACAGGAUGAATT-3′, R: 5′-UUCAUCCUGUCCUUGAGGCTT-3′) targeting the *IFNB1* gene was used to knock down *IFNB1*, and a scrambled siRNA (SiNC: F: 5′-UUCUCCGAACGUGUCACGUTT-3′, R: 5′-ACGUGACACGUUCGGAGAATT-3′) was used as the control. The MARC-145 cells in the 12-well plate were cultured to 30–50% confluency. Then, the siRNA fragments at 40 pmol were transfected into cells for gene knockdown using Lipofectamine 2000 (Invitrogen, Carlsbad, CA, USA), according to the manufacturer’s instructions. The cells were harvested at 24 h after transfection, and the efficiency of RNA interference was evaluated using qPCR.

### 2.7. Western Blotting Analysis

The MARC-145 cells or Vero cells were washed with cold PBS and lysed in RIPA Lysis or Extraction Buffer (Thermo Fisher Scientific, Waltham, MA, USA). The protease inhibitor (PMSF, 10%) was added to block the endogenous proteolysis. The cell lysates were centrifuged at 14,000× *g* for 20 min at 4 °C; the supernatant was collected. The protein concentration was determined using a BCA Protein Quantification Kit (Thermo Fisher Scientific, Waltham, MA, USA). An equal amount of protein samples were subjected to SDS-PAGE, transferred to an NC membrane, and then blocked with 5% skimmed milk in PBST buffer for 1 h at room temperature. Subsequently, anti-AEN (1:2000), anti-PEDV-N (1:50,000), anti-Flag (1:5000), and anti-β-Actin (1:5000) were used as the primary antibodies at 4 °C overnight. After being washed with PBST, the membrane was incubated with the horseradish peroxidase-conjugated goat anti-rabbit, or goat anti-mouse IgG (1:8000) for 1 h at room temperature. The membrane was incubated with ECL regent (Thermo Fisher Scientific, Waltham, MA, USA) and chemiluminescence was detected with the ChemiDoc touch imaging system (Bio-Rad, Hercules, CA, USA).

### 2.8. Statistical Analysis

All experiments were performed independently thrice, and data are presented as means ± SEM. The two-tailed Student’s *t*-test or two-way ANOVA was used for statistical analysis. Any *p* values < 0.05 were considered statistically significant.

## 3. Results

### 3.1. An Increase in Virus Replication Inhibited AEN Production during PEDV Infection in MARC-145 Cells

**Figure 1:**
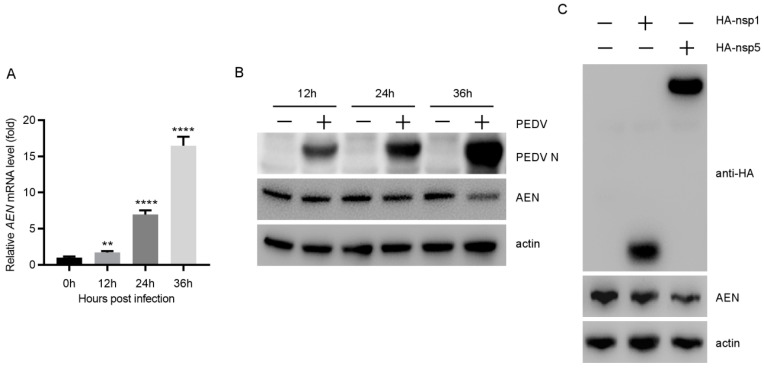
The detection of AEN mRNA levels by qPCR and AEN production using Western blot. (**A**) MARC-145 cells were infected with PEDV (85-7) at an MOI of 0.1 and harvested at the indicated times. The AEN mRNA levels were analyzed using qPCR. (**B**) The production of AEN and PEDV N proteins were tested by Western blot analyses. Actin was used as the sample-loading control. (**C**) MARC-145 cells were transfected with HA-nsp1, HA-nsp5, or the empty vector (1.5 μg). At 24 h post-transfection, the cells were harvested and protein expression was analyzed with Western blotting (** *p* < 0.01, **** *p* < 0.0001).

A transcriptome approach was employed to screen the potential antiviral proteins of MARC-145 cells in our previous work [11]. We found that the *AEN* gene was upregulated in MARC-145 cells during PEDV infection. AEN (apoptosis-enhancing nuclease) is a closely related member of ISG20 from the DEDDh exonuclease family in human [18]. Many studies have confirmed the antiviral activity of ISG20 in RNA virus infection, suggesting that AEN may play a similar role during PEDV infection. We identified the characteristics of AEN in expression and production during PEDV infection. The mRNA levels of AEN were significantly upregulated 1.7-, 6.9-, and 16.5-fold at 12, 24, and 36 h post infection, respectively, compared with that of uninfected MARC-145 cells (Figure 1A). AEN production was steady in the MARC-145 cells at 12 hpi, but was significantly downregulated at 24 and 36 hpi (Figure 1B). It should be noted that the virus replication gradually increased from 12 to 36 hpi, suggesting that the higher virus titers may cause AEN degradation via an unknown mechanism. Subsequently, the eukaryotic expression plasmid HA-nsp1 or HA-nsp5 was transfected into the MARC-145 cells, and it was found that overexpression of HA-nsp1 and HA-nsp5 significantly decreased AEN protein levels compared with an empty vector (Figure 1C). These results indicate that PEDV infection could activate the AEN expression, while overall increased virus replication downregulated AEN production via nsp1 and nsp5.

### 3.2. AEN Overexpression Inhibited PEDV Replication in MARC-145 Cells

**Figure 2:**
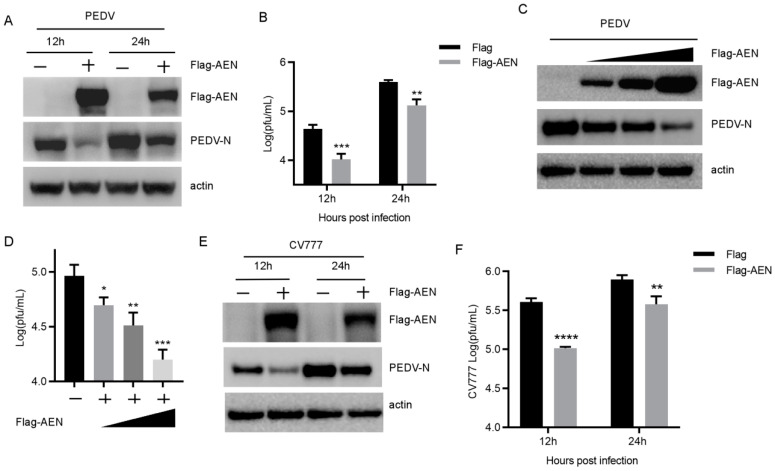
AEN inhibited PEDV replication in MARC-145 cells. (**A**,**E**) MARC-145 cells were transfected with plasmid Flag-AEN or the empty vector. At 24 h post-transfection, the cells were infected with PEDV (85-7 and CV777) at an MOI of 0.1 and harvested at the indicated times. Flag-AEN and PEDV N protein expression was analyzed with Western blotting. Actin was used as the sample-loading control. (**B**,**F**) PEDV titers in the culture supernatants of the MARC-145 cells treated described in (**A**,**E**) were measured as PFUs. (**C**) MARC-145 cells were transfected with plasmid Flag-AEN 1 μg, 1.5 μg, 2 μg, or empty vector. At 24 h post-transfection, the cells were infected with PEDV (85-7) at an MOI of 0.1 and harvested at 12 hpi. Flag-AEN and PEDV N protein expression were analyzed with Western blotting. Actin was used as the sample-loading control. (**D**) PEDV titers in the culture supernatants of the MARC-145 cells treated described in (**C**) were determined by PFUs (* *p* < 0.05, ** *p* < 0.01, *** *p* < 0.001, **** *p* < 0.0001).

To investigate whether AEN could regulate PEDV replication, the MARC-145 cells were transfected with Flag-AEN and empty vector, and then infected with PEDV 85-7 at an MOI of 0.1. The cells and supernatants of the infected cells were harvested at the indicated time points to assay the PEDV viral titers. The results show that the production of PEDV N protein significantly decreased upon AEN overexpression at 12 and 24 h post-infection (Figure 2A). Meanwhile, the viral titers in the supernatants transfected with Flag-AEN were significantly lower (4.2- and 2.9-fold) than those in the cells transfected with empty vector (Figure 2B). MARC-145 cells were then transfected with increased doses of Flag-AEN before virus infection and the inhibitory effects on PEDV N protein production and viral titers were observed in a dose-dependent manner (Figure 2C,D). In addition, we also verified the impact of AEN on the replication of the other PEDV strain, and the results showed that overexpression of AEN could also inhibit the replication of PEDV CV777 (Figure 2E,F).

### 3.3. The Antiviral Effect of AEN Was Independent of Its Exonuclease Activity 

**Figure 3:**
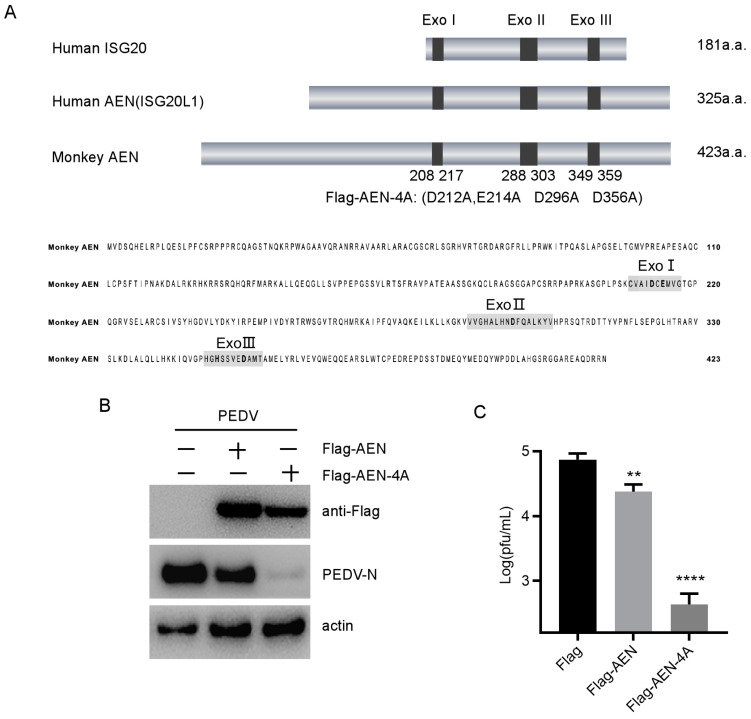
AEN and AEN-4A overexpression inhibited PEDV replication in MARC-145 cells. (**A**) Schematic presentation of amino acid length of human ISG20, human AEN, and monkey AEN. Gray represents the total length of the protein, and the dark gray represents three exonuclease domains. The Exo I, Exo II, and Exo III domains of monkey AEN are amino acid residues 208 to 217, 288 to 303, and 349 to 359, respectively. (**B**) MARC-145 cells were transfected with plasmid Flag-AEN, Flag-AEN-4A, or empty vector. At 24 h post-transfection, the cells were infected with PEDV at an MOI of 0.1 and harvested at 12 hpi. Flag-AEN, Flag-AEN-4A, and PEDV N protein expression was analyzed with Western blotting. Actin was used as the sample-loading control. (**C**) PEDV titers in the culture supernatants of the MARC-145 cells treated as described in (**B**) were measured as PFUs (** *p* < 0.01, **** *p* < 0.0001).

Alignment of protein sequences of the monkey AEN, human AEN, and human ISG20 showed that the monkey AEN also contains the same three exonuclease motifs (Exo I–III) and five conserved amino acids (Figure 3A). ISG20 can inhibit replication of several RNA viruses dependent on its exonuclease activity [18]. These findings suggest that AEN may have the potential to inhibit PEDV (an RNA virus) replication via its exonuclease function. Since DEDD residues are known to be essential for nuclease activity, point mutations of D212A, E214A, D296A, and D356A were introduced into the catalytic center of Exo I–III to generate AEN mutant Flag-AEN-4A (Figure 3A). AEN and AEN-4A were overexpressed in MARC-145 cells for 24 h, and the cells and the supernatants were harvested to determine the virus yield at 12 hpi. The results showed that the production of PEDV N protein and the virus titers were obviously downregulated upon overexpressing AEN and AEN-4A in MARC-145 cells (Figure 3B,C). Unexpectedly, nuclease-activity-deficient AEN-4A showed 100-fold higher antiviral activity than wild-type AEN (Figure 3C). These data suggest that the antiviral activity of AEN was independent of its exonuclease activity, and its exonuclease activity might hamper its antiviral activity.

### 3.4. AEN and AEN-4A Increase the Transcription of Type I IFN and ISGs

**Figure 4:**
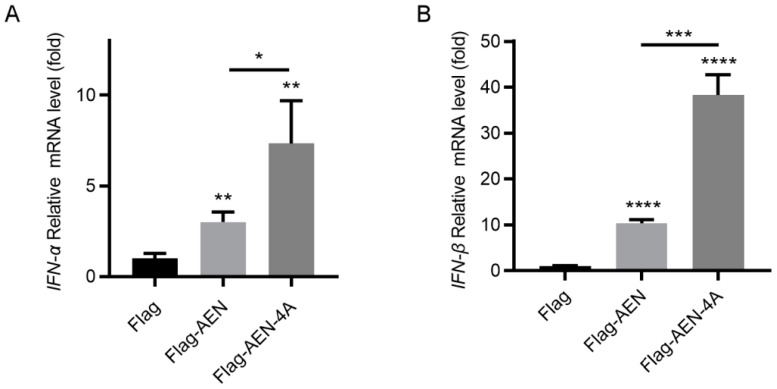
Effects of AEN and AEN-4A on type I IFN in MARC-145 cells. MARC-145 cells were transfected with empty vector, Flag-AEN, or FLAG-AEN-4A plasmid, and after 24 h, cell samples were collected to detect INF-α (**A**) and IFN-β (**B**) transcription levels using a qPCR assay. * *p* < 0.05, ** *p* < 0.01, *** *p* < 0.001, **** *p* < 0.0001.

**Figure 5:**
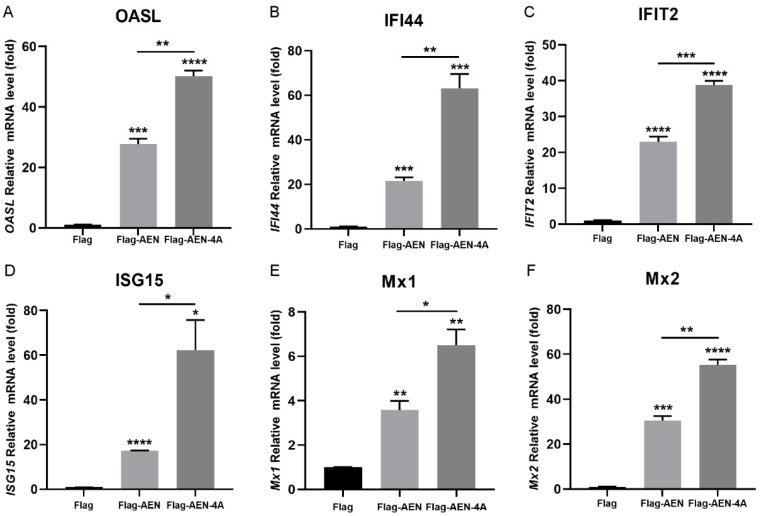
AEN and AEN-4A induced partial ISG expression in MARC-145 cells. MARC-145 cells were transfected with empty vector, Flag-AEN, or FLAG-AEN-4A plasmid, and after 24 h, the transcription levels of OASL (**A**), IFI44 (**B**), IFIT2 (**C**), ISG15 (**D**), Mx1 (**E**), and Mx2 (**F**) were detected using qPCR. * *p* < 0.05, ** *p* < 0.01, *** *p* < 0.001, **** *p* < 0.0001.

In view of the fact that both AEN and ISG20 belong to the DEDDh exonuclease family proteins, and ISG20 inhibits viral replication by activating the type I IFN immune responses [22,25], we therefore speculated whether AEN also exerts its antiviral activity through the IFN pathway, since our previous study also proved that IFN-β can inhibit PEDV replication [10]. After overexpression of AEN and AEN-4A for 24 h, the transcriptional expression levels of type I IFN were detected. The results showed that overexpression of AEN and mutant AEN-4A can significantly induce the transcription of IFN-α and IFN-β, and the level of type I IFN induced by AEN-4A was significantly higher than that of AEN (Figure 4). These results suggest that AEN regulates the expression of type I IFN, and the exonuclease activity may limit the regulation of AEN to type I IFN. AEN and AEN-4A have been shown to induce IFN-α and -β expression, but whether they activate the type I IFN response pathway remains unclear. Therefore, we used a qPCR assay to detect the transcription levels of six ISGs (OASL, IFI44, IFIT2, ISG15, Mx1, Mx2), which have been reported to inhibit PEDV replication in our previous studies [11]. The results showed that both AEN and AEN-4A could induce upregulation of these ISGs, and the induction level of AEN-4A was significantly higher than that of AEN (Figure 5). These results demonstrate that AEN and AEN-4A can activate the type I IFN response pathway and suggest that AEN protein may act as an upstream molecule in the type I IFN-mediated antiviral signaling pathway.

### 3.5. AEN and AEN-4A Exert Antiviral Effects Dependent on IFN

**Figure 6:**
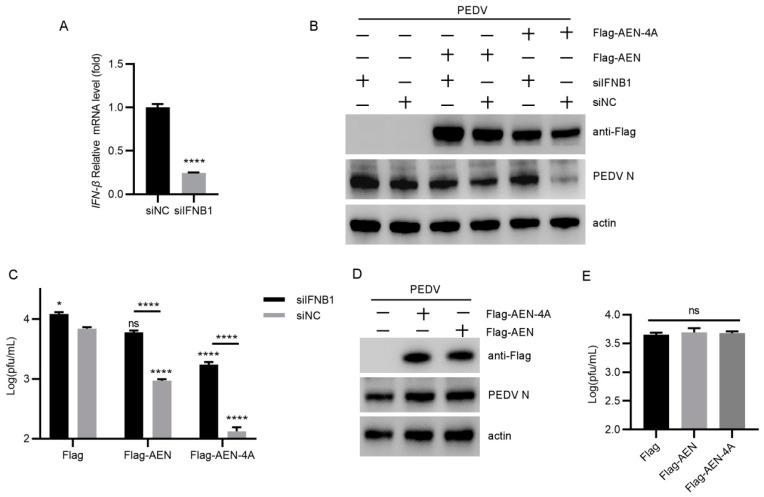
AEN and AEN-4A exert antiviral effects through IFN. (**A**) MARC-145 cells were simultaneously transfected with siNC and poly I:C or siIFNB1 and poly I:C, respectively. After 24 h transfection, cell samples were collected and the transcription levels of IFNB1 were detected using a qPCR assay; (**B**) MARC-145 cells were co-transfected with siIFNB1 and Flag, flag-AEN, or FLAG-AEN-4A, respectively, and negative controls were set up to co-transfect siNC and Flag, flag-AEN, or flag-AEN-4A, respectively. After 24 h transfection, the PEDV 85-7 strain with MOI = 0.1 infected MARC-145 cells. The cell samples and cell supernatant were collected at 12 hpi, and the PEDV N protein levels (**B**) were detected using a Western blot test, and the viral titers (**C**) of the cell supernatant was determined using a PFUs test, respectively; Vero cells were transfected with Flag, flag-AEN, and flag-AEN-4A, respectively, for 24 h, and then Vero cells were infected with PEDV 85-7 strain with MOI = 0.1 for 12 h. The cell samples and supernatant were collected and analyzed by Western blot assay to detect PEDV N protein expression levels (**D**), and a PFU assay was used to determine viral titers in the supernatant (**E**) (* *p* < 0.05, **** *p* < 0.0001, ns means not significant).

To further clarify whether the inhibitory effect of AEN was dependent on IFN response, we used siRNA to knock down the expression level of IFN-β. The results showed that transfection of siRNA (siIFNB1) targeting IFNB1 significantly decreased the expression of IFN-β compared with the control siRNA (siNC) (Figure 6A). Subsequently, we co-transfected siRNA with AEN or AEN-4A eukaryotic expression vector, and the results showed that IFN-β interference could significantly increase the viral N protein expression and viral titer (Figure 6B,C). Meanwhile, when INF-β is knocked down and AEN or AEN-4A is overexpressed, the antiviral activity of AEN is almost lost, while the antiviral activity of AEN-4A is weakened (Figure 6B,C). Considering that Vero cells are also African green monkey kidney cells and a good cell model for the in vitro study of PEDV [26], and most importantly, they are type I IFN-deficient cells [27], AEN and AEN-4A were overexpressed in Vero cells to verify the antiviral effect after infection with PEDV. The results showed that AEN and AEN-4A had no antiviral effect on Vero cells (Figure 6D,E). These results indicate that the antiviral effects of AEN and AEN-4A were dependent on IFN.

## 4. Discussion

PEDV has led to the outbreak of PED and its rapid spread in the world in the past few decades, and there have been a wide variety of mutations [28,29,30]. In the process of interactions between virus and host, viruses have evolved a variety of strategies to evade the innate immune response [31,32]. With the continuous emergence and recurrence of PEDV, it is crucial to excavate antiviral proteins and explore antiviral therapies or effective vaccines.

AEN was found to increase apoptosis after exposure to ionizing radiation. It possesses DNA- and RNA-cleaving Exo I–III exonuclease domains. Meanwhile, it will likely promote apoptosis in response to ionizing radiation and other stimuli, such as staurosporine [12]. Meanwhile, it is shown that AEN targets both single- and double-stranded DNA and RNA in human [13]. In this study, AEN was confirmed as an antiviral component, and its expression was significantly upregulated in MARC-145 cells infected with PEDV. In contrast, the amount of AEN protein decreased during the progression of the virus replication, suggesting that the virus may inhibit AEN’s antiviral activity by degrading the AEN protein, or inhibiting AEN protein synthesis. These findings suggest that PEDV has developed the strategies to reduce the expression of AEN protein to evade its antiviral activity. Numerous studies have demonstrated that PEDV can adopt strategies to evade innate immunity. For instance, PEDV inhibits interferon signaling by targeting the degradation of STAT1 via the ubiquitin proteasome system [33]. By cleaving NEMO (NF-κB essential modulator), PEDV nsp5, a 3C-like protease, prevents type I IFN signaling [34]. PEDV nsp1 interrupts the binding of CREB-binding protein (CBP) to IRF3 by degrading CBP in a proteasome-dependent manner, thereby inhibiting the production of type I interferon [35]. Furthermore, three motifs (amino acids 67 to 71, 78 to 85, and 103 to 110) of PEDV nsp1 create a stable functional region for inhibiting protein synthesis [36]. PLP2, one of the two papain-like protease domains (PLP1 and PLP2) of nsp3, antagonizes host innate immune response by cleaving the interferon-stimulated gene product 15 (ISG15) [37]. Shi et al. [38] found that nsp5, cleaving porcine gasdermin D (pGSDMD) at the Q193-G194 junction, inhibited pGSDMD-mediated pyroptosis, and thus facilitated viral replication during the initial period. In this study, we have confirmed that nsp1 and nsp5 can mediate the reduction of AEN protein during PEDV replication. Whereas further study is needed to determine which pathway they depend on to decrease AEN production, and which host or viral proteins are involved.

**Figure 7:**
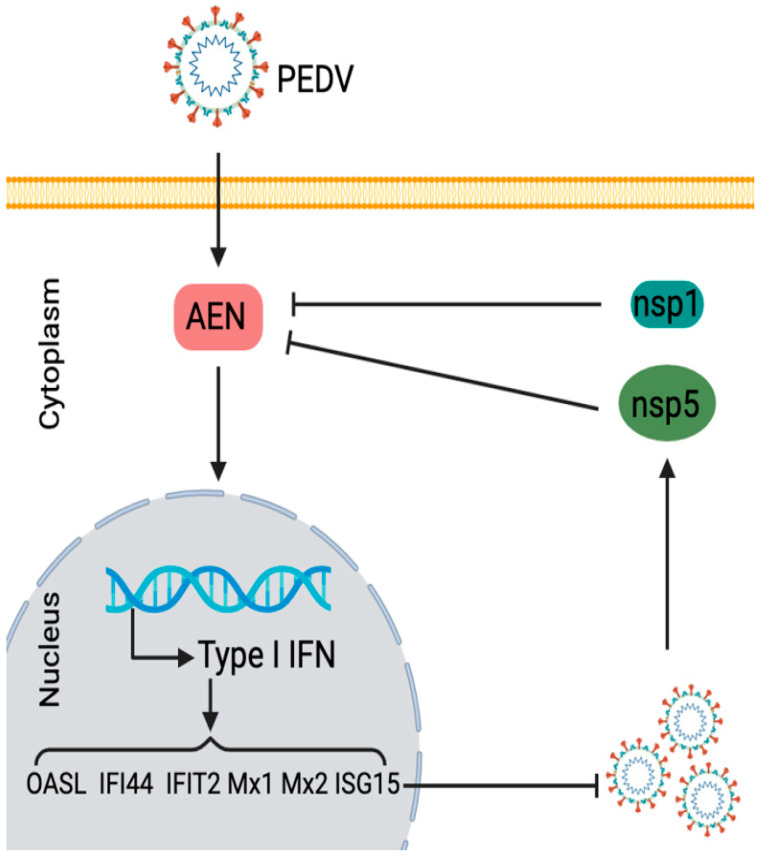
The pathogenicity in the relations between PEDV infection, IFN, and AEN.

Meanwhile, relevant studies have confirmed that the antiviral effect of ISG20 proteins depends on its exonuclease function [18]. However, after overexpression of AEN and AEN-4A, we were surprised to find that AEN-4A had a better antiviral effect, and the key amino acids of exonuclease even hindered the antiviral effect of AEN. It has been reported that ISG20 can enhance type I IFN production and upregulate ISGs, thus inhibiting viral replication. Our previous studies confirmed that PEDV inhibits the expression of IFN-β during virus infection, and the presence of INF-β inhibits PEDV replication [10]. We then speculated whether AEN might induce an increase in IFN production. The results demonstrated that both AEN and AEN-4A could significantly induce an increase in the transcription level of IFN-α and -β, and the induction ability of AEN-4A was higher than that of AEN, which could also explain why the antiviral effect of AEN-4A was significantly higher than that of AEN. The production of type I IFN is known to activate the transcription levels of many ISGs through the JAK/STAT pathway, thereby acting as an antiviral agent [39]. Therefore, we selected six ISGs (OASL, ISG15, IFIT2, IFI44, Mx1, and Mx2) that could inhibit PEDV replication to verify whether AEN and AEN-4A can promote an increase in their transcription levels. As expected, both AEN and AEN-4A can promote their expression levels, and AEN-4A induces significantly higher levels than AEN. It is worth noting that transfection of IFNB1-specific siRNA could counteract the inhibitory effect of AEN and AEN-4A on PEDV replication, and the antiviral effect was completely lost in IFN-deficient Vero cells. Combining the above findings suggests that AEN protein inhibits PEDV replication through the antiviral pathway mediated by type I IFN, and PEDV mediates the decrease of AEN production through nsp1 and nsp5 to evade immune response (Figure 7).

The above results also suggest that the increase in type I IFN and ISGs induced by AEN may be involved in the upstream regulation of the type I IFN signaling pathway. Typically, cellular-pattern-recognition receptors (PRRs) [40,41] recognize the corresponding pathogen-associated molecular patterns (PAMPs) and thus lead to the activation of transcription factors such as IRFs and NF-κB [42] through a signaling cascade, which in turn is imported into the nucleus to induce IFN production [43]. Therefore, it is still necessary to explore which proteins in the IFN signaling pathway are regulated by AEN and AEN-4A to activate the IFN immune response, and how they are regulated.

In another study, the ability to activate type I IFN was lost after the mutation of key amino acids in the Exo II region of ISG20 [25], while in our study, the ability to activate type I IFN was significantly increased after the mutation of four key amino acids in three nuclease functional regions of AEN. This suggests that these four amino acids play a key role in activating the IFN immune response, or that non-homologous regions with ISG20 also have corresponding functions. It was found that the N-terminal region of human AEN (aa 1–109) is involved in binding with DAPK1 and p53 upon arsenite exposure [44], while this region was also present in monkey AEN, but not in human ISG20. Therefore, the ability of AEN to activate the IFN immune response may be related to the interaction between this region and other proteins. In addition, there are few studies on human AEN and almost none on monkey AEN, so more studies are needed to discuss its biological characteristics.

## Data Availability

The data presented in this study are available in this article and supplementary materials.

## Supplementary Materials

The following supporting information can be downloaded at: https://www.mdpi.com/article/10.3390/pathogens13010024/s1, Table S1: Primers used in constructing plasmids; Table S2: Primers used in qPCR.
