# Supplementary material for: AEN Suppresses the Replication of Porcine Epidemic Diarrhea Virus by Inducing the Expression of Type I IFN and ISGs in MARC-145 Cells
Source: Pathogens. 2023 Dec 27;13(1):24. doi: 10.3390/pathogens13010024 (PMC10819003; doi:10.3390/pathogens13010024)
Supplement: Supplementary file 1 [file pathogens-13-00024-s001.zip › pathogens-2778846-supplementary.pdf]

Table S1. Primers used in constructing plasmids

Name	Sequence (5'-3')
Flag-AEN-F	CGACGAATTCGCCACCATGGTTGACTCGCAGCACGAGT
Flag-AEN-R	GATGGGATCCATTCCTTCTGTCCTGTGCCTCC
HA-nsp1-F	GCACGAATTCATGGCTAGCAACCATGTCACA
HA-nsp1-R	GCACCTCGAGCTAACCACCACGACGACCAAAAGT
HA-nsp5-F	GCACGAATTCATGGCTGGCTTGCGTAAGATGGCA
HA-nsp5-R	GCACCTCGAGCTACTGAAGATTAACGCCATACAT
AEN D212A/E214A-F	CCAGCAAGTGTGTGGCTATCGCCTGTGCGATGGTGGGCACGGGACCCCAAGG
AEN D212A/E214A-R	CCTTGGGGTCCCGTGCCCACCATCGCACAGGCGATAGCCACACACTTGCTGG
AEN D296A-F	GGGGCACGCGCTGCACAACGCCTTCCAGGCCCTCAAGTATG
AEN D296A-R	CATACTTGAGGGCCTGGAAGGCGTTGTGCAGCGCGTGCCCC
AEN D356A-F	GGGCACTCATCAGTAGAAGCTGCCATGACAGCCATGGAGC
AEN D356A-R	GCTCCATGGCTGTCATGGCAGCTTCTACTGATGAGTGCCC

Table S2. Primers used in qPCR

Name	Sequence (5'-3')
AEN-F	GCCCCGTTGGAAGATTAC
AEN-R	GGCATTTGGGATGGTGAA
IFN-α-F	GGCACTGCCCTTTGCTTTAC
IFN-α-R	GTTATCCAGGCTGTGGGTCTCA
IFN-β-F	GCAATTGAATGGAAGGCTTGA
IFN-β-R	CAGCGTCCTCCTTCTGGAACT
OASL-F	AAAGAGAGGCCCATCATCCT
OASL-R	ATCTGGGTAACCCCTCTGCT
ISG15-F	GGTGTCAGAGCTGAAGGCAAAG
ISG15-R	TCCACCACCAGCAGGACCGT
IFI44-F	AAGGGAGTTGATAAACGCTG
IFI44-R	GAATAATTGCTAACCACCGA
IFIT2-F	CTGGTCACCTGGGGAAACTA
IFIT2-R	GAGCCTTCTCAAAGCACACC
Mx1-F	TTGAGGACCACCCACATTTCA
Mx1-R	5'-TTCTAACAGCGGCAGAGATTTAC-3'
Mx2-F	5'-CTTTCAAACGCATCCATGTTTC-3'
Mx2-R	5'-GGTGGCTCTCCCTTATTTGTTCT-3'
β-actin-F	5'-CGGGAAATCGTGCGTGAC-3'
β-actin-R	5'-ATGCCCAGGAAGGAAGGTTG-3'
